# Mathematical learning difficulties subtypes classification

**DOI:** 10.3389/fnhum.2014.00057

**Published:** 2014-02-10

**Authors:** Giannis Karagiannakis, Anna Baccaglini-Frank, Yiannis Papadatos

**Affiliations:** ^1^Department of Primary Education, Research Center of Psychophysiology and Education, National and Kapodistrian University of AthensAthens, Greece; ^2^Department of Education and Human Sciences, University of Modena and Reggio EmiliaReggio Emilia, Italy

**Keywords:** core deficits, dyscalculia, internal representation of numbers, mathematical learning difficulties, multi-deficit model, number sense

## Introduction

Mathematics is a complex subject including different domains such as arithmetic, arithmetic problem solving, geometry, algebra, probability, statistics, calculus, … that implies mobilizing a variety of basic abilities associated with the sense of quantity, symbols decoding, memory, visuospatial capacity, logics, to name a few. Students with difficulties in any of the these abilities or in their coordination, may experience mathematical learning difficulties. Understanding the cognitive nature of the various mathematical domains, as well as the mechanisms mediating cognitive development, has fascinated researchers from different fields: from mathematics education to developmental and cognitive psychology and neuroscience.

The field of cognitive psychology has a long history in the studies of cognitive difficulties involved in developing the representation and learning general use of numbers in mathematics (e.g., Campbell, 2005). However, as Fletcher et al. (2007) note, there are “no consistent standards by which to judge the presence or absence of LDs [learning difficulties] in math” (p. 207), and there is still disagreement concerning the question of a definition, operational criteria, and prevalence (Lanfranchi et al., 2008; Mazzocco, 2008). In general, the term Mathematical Learning Difficulty (MLD) is used broadly to describe a wide variety of deficits in math skills, typically pertaining the domains of arithmetic and arithmetic problem solving. We will use MLDs to refer to learning difficulties in these domains as well as other mathematical domains like the ones mentioned above.

Within the field of mathematics education, many frameworks and theories have been developed to analyze teaching and learning processes and difficulties involved with these and other mathematical tasks (e.g., Freudenthal, 1991; Schoenfeld, 1992, 2011; Bharath and English, 2010). Recently, the field has shown interest in perspectives from cognitive neuroscience (e.g., Grabner and Ansari, 2010).

Although developmental and classification models in these fields have been developed (for example, Geary and Hoard, 2005; Desoete, 2007; von Aster and Shalev, 2007), to our knowledge, no single framework or model can be used for a comprehensive and fine interpretation of students' mathematical difficulties, not only for scientific purposes, but also for informing mathematics educators. As mathematics educators^1^, we believe that reaching a model that combines existing hypotheses on MLD, based on known cognitive processes and mechanisms, could be used to provide a *mathematical profile* for every student.

Our aims with this contribution are to: (1) provide an overview of the most relevant hypotheses in the present day's literature regarding possible deficits that lead to MLD and of possible classifications of MLD subtypes; (2) and to build on such literature, using a multi-deficit neurocognitive approach, to propose a classification model for MLD describing four basic cognitive domains within which specific deficits may reside.

In order to reach our first objective we will describe the current hypotheses on neurocognitive deficits that may lead to MLD specifically related to numbers, and then we will provide examples of the most relevant classifications of MLD, based on a possible deficit in basic cognitive functions.

## Hypotheses for difficulties in the learning of numbers

Certainly deficits that lead to difficulties in processing numbers are of primary importance among the hypotheses for MLD. The literature refers to two preverbal or non-symbolic systems for processing quantities: (1) the object tracking system (OTS) that is precise, limited by its absolute set size, and that creates an *object file* with concrete information for each objects observed simultaneously (Brannon and Roitman, 2003; Xu, 2003; Fayol and Seron, 2005; van Herwegen et al., 2008; Cordes and Brannon, 2009; Cantlon et al., 2010; Piazza, 2010); (2) the approximate number system (ANS) that is extensible to very large quantities, operates on continuous dimensions, and yields and approximate evaluation in accordance with Weber's law (Xu and Spelke, 2000; Mix et al., 2002; Halberda and Feigenson, 2008; Piazza, 2010).

The main hypotheses based on deficits in these systems and other mechanisms specific to numerical processing have been reviewed by Andersson and Östergren (2012), and classified into the following categories:

- defective ANS;
- defective OTS;
- defective numerosity-coding;
- access deficit;
- multiple deficit.

## Classifications of MLD and the hypothesis of domain general cognitive deficit

As noted in the introduction, acquiring basic mathematical skills requires possessing, building and promoting a range of abilities. The core systems of number, seem to be quite important in understanding the nature of the development of numerical cognition, but these are not the only systems upon which success in mathematics lies. Many researchers have attempted to describe subtypes in MLD (Geary, 1990; Rourke, 1993; Fuchs and Fuchs, 2002; Geary, 2004; Geary and Hoard, 2005). Geary was one of the first who tried to connect “Mathematics Disorder” with neuropsychological deficits (Geary, 1994). He posited three key subtypes of deficits (confirmed in Geary and Hoard, 2005):

- *procedural* (left hemisphere), in which children present a delay in acquiring simple arithmetic strategies, which may be a result of verbal working memory deficits, but also deficits in conceptual knowledge.
- *semantic memory* (left hemisphere), in which children show deficits in retrieval of facts because of a long term memory deficit.
- *spatial* (right hemisphere), in which children show deficits in the spatial representation of number.

In general, Geary's classification and the others proposed in the literature (for a review see: Desoete, 2004, 2007; Stock et al., 2006) lead to the identification of the 3 subtypes listed, as well as, one based on a *number knowledge* deficit. However such classifications are not completely satisfactory because in general the profiles of the children met in practice do not appear to belong to any subtype, but instead be constituted of several characteristics pertaining different subtypes (Desoete, 2007).

We believe this to be the case, because the subtypes are not characterized by basic cognitive processes, such as working memory (WM), long term memory (semantic memory), executive functions, fact retrieval and, by extension, calculation and fluency. Together with the existing hypotheses of domain specific deficits in number processing that we have presented in section Hypotheses for Difficulties in the Learning of Numbers, we support an additional hypothesis of a *domain general cognitive deficit* underlying MLD (Geary, 2004; Geary and Hoard, 2005), which emanates from converging evidence showing that such cognitive functions are involved in mathematical performance in both adults and children (Fuchs et al., 2005; Andersson, 2007, 2008; Swanson et al., 2008).

## Classification model of mathematical learning difficulties

Taking into account the literature presented as well as unpublished clinical observations, we propose a classification model for MLD (Table 1) describing four basic cognitive domains within which specific deficits may reside. For each subtype we also list specific systems involved and typical mathematical difficulties.

**Table 1:** **Classification model for MLD, proposing 4 subtypes, possible specific systems involved, and typical mathematical difficulties encountered**.

**Subtype**	**Specific systems involved**	**Mathematical difficulties^1^**
1. Core number	Internal representation of quantity: ANS OTS Numerosity-Coding representation of symbols Access deficit	Arithmetical domain: Basic sense of numerosity (Butterworth, 2005), and estimating accurately a small number of objects e.g., 4–5 (subitizing) (Butterworth, 2010; Piazza, 2010) Estimating approximately different quantities (Piazza et al., 2010) Placing numbers on number lines, SNARC effect (Zorzi et al., 2002, 2005; Menon et al., 2000; Siegler and Opfer, 2003) Managing Arabic symbols (Ansari et al., 2006; Rousselle and Noël, 2007) Transcoding a number from one representation to another (analog-Arabic-verbal) (Wilson and Dehaene, 2007) Grasping the basic counting principles (Gallistel and Gelman, 1992; Geary et al., 1996; Geary and Hoard, 2005) Capturing the meaning of place value (including in decimal notation) (Russell and Ginsburg, 1984; Geary, 1993); Capturing the meaning of the basic arithmetic operation symbols (+, −, ×, :).
2. Memory (retrieval and processing)	Working memory^2^ (WM) Inhibition of irrelevant information from entering WM Semantic memory	All mathematical domains: Retrieving numerical facts (Geary, 1993, 2004; von Aster, 2000; Woodward and Montague, 2002) Decoding—confusing terminology (numerator, denominator, isosceles, equilateral,…) (Geary, 1993; Hecht et al., 2001) Transcoding verbal rules or orally presented tasks (Rourke and Finlayson, 1978; Rourke, 1993; Brysbaert et al., 1998; Andersson, 2007; Swanson et al., 2008) Performing mental calculation accurately (Campbell, 1987a,b, 1991; Ashcraft, 1992; Andersson and Östergren, 2012) Remembering and carrying out procedures as well as rules and formulas (Pellegrino and Goldman, 1987; Gerber et al., 1994) (Arithmetic) problem solving (keeping track of steps) (Jitendra and Xin, 1997; Passolunghi and Siegel, 2001, 2004; Fuchs and Fuchs, 2002, 2005; Andersson, 2007; Swanson et al., 2008).
3. Reasoning	Various executive mechanisms: Entailment Inhibition (not connected to WM) Updating relevant information, shifting from one operation-strategy to another Updating and strategic planning Decision-making	All mathematical domains: Grasping mathematical concepts, ideas and relations (Schoenfeld, 1992; Geary, 1993) Understanding multiple steps in complex procedures/algorithms (Russell and Ginsburg, 1984; Bryant et al., 2000; Geary, 2004) Grasping basic logical principles (conditionality—“if… then… ” statements—commutativity, inversion,…) (Núñez and Lakoff, 2005) Problem solving (decision making) (Schoenfeld, 1992; Desoete and Roeyers, 2006).
4. Visual- Spatial	Visuo-spatial (VS) WM^3^, Visuo-spatial reasoning/perception	Domains of written arithmetic, geometry, algebra, analytical geometry, calculus: (Geary, 1993, 2004; Rourke and Conway, 1997; Venneri et al., 2003; Mammarella et al., 2010) Interpret and use spatial organization of representations of mathematical objects (for example, numbers in decimal positional notation, exponents, or geometrical figures) Placing numbers on a number line (Cooper, 1984; Dehaene and Cohen, 1997) Recognizing Arabic numerals and other mathematics symbols (confusion in similar symbols) (Venneri et al., 2003) Written calculation, especially where position is important (e.g., borrowing/carrying) (Heathcote, 1994; Mammarella et al., 2010; Szucs et al., 2013) Controlling irrelevant visuo-spatial information (Mammarella and Cornoldi, 2005; Mammarella et al., 2013) Visualizing and analyzing geometric figures (or subparts of them), in particular visualizing rigid motions such as rotations (Thompson et al., 2013) Interpreting graphs, understanding and interpreting when the math information are organized visual-spatially (tables)^4^.

1 *These can also be read as “mathematical skills” if the model is being used to identify the student's stronger specific systems*.

2 *In particular the phonological WM used in selecting verbal over spatial information as relevant (for e.g., De Smedt et al., 2010)*.

3 *There is increasing evidence showing that many of these difficulties may be related, but not limited, to deficits in VSWM (Heathcote, 1994; Cornoldi et al., 1999; Kyttälä et al., 2003; Mammarella et al., 2006, 2010)*.

4 *Difficulties of type 4.7 are well known in the mathematics education literature, but we are not aware of studies that relate these to basic cognitive abilities*.

An analysis of a student's performance on a set of well-designed tasks can lead to the singling out of specific areas of difficulty (and strength), by comparing the specific systems and difficulties implied in each incorrect (or correct) answer to the tasks. The student's profile will be then described as a single subtype or as a combination of subtypes in which types of mathematical tasks and possible deficits to specific systems are highlighted.

## Conclusions

We conclude highlighting the innovative value of the present etiological model. First, we may refer to it as being *multidimensional* in that it proposes a transition from the one-dimensional “Dyscalculia” to the multidimensional “Mathematical Learning Difficulties,” bringing into the picture mathematical domains other than the ones typically considered by the MLD literature until today. This leads to the second important feature: the model has direct implications for the field of mathematics education and may become an important tool for educators involved both in primary or secondary education. This is because the model allows to identify mathematical profiles of students early on, and these can be used to design more effective and comprehensive intervention programs, focusing on the students' strengths to compensate weaknesses and provide motivation. In fact, we believe, that in general intervention should focus mostly on the students' strengths, because this can have positive effects on motivation, while attempts to address students' weaknesses directly are likely to contribute to de-motivation and further failures. Moreover, educators, from researchers to teachers, can use the model to easily create tasks for working with their students.

Finally, the suggested classification model of MLD is expected to be clinically useful, as has been observed during a first study of its validity, the results of which will be soon submitted for publication. However we note that the model is at an early phase of development, and it can and will be improved through further research.

## Footnotes

^1^The authors hold graduate and post graduate degrees in mathematics and mathematics education.
